# Improving cell survival and engraftment in vivo via layer-by-layer nanocoating of hESC-derived RPE cells

**DOI:** 10.1186/s13287-020-01986-z

**Published:** 2020-11-25

**Authors:** Liyan Ru, Nan Wu, Keyu Wei, Yuxiao Zeng, Qiyou Li, Chuanhuang Weng, Chunge Ren, Bangqi Ren, Da Huo, Yijian Li, Xisu Hu, Zuoxin Qin, Yajie Fang, Chuhong Zhu, Yong Liu

**Affiliations:** 1 Department of Ophthalmology, Key Lab of Visual Damage and Regeneration & Restoration of Chongqing, Southwest Hospital, Chongqing, 400038 China; 2 Department of Anatomy, Key Lab for Biomechanics and Tissue Engineering of Chongqing, State Key Laboratory of Trauma, Burn and Combined injury, Department of Plastic and Aesthetic Surgery, Southwest Hospital, Army Medical University (Third Military Medical University), Chongqing, 400038 China

**Keywords:** Retinal pigment epithelial, Tissue engineering, Encapsulation, Retinal degenerative diseases

## Abstract

**Background:**

Human embryonic stem cell-derived retinal pigment epithelial (hESC-RPE) cell transplants have served as a cell therapy for treating retinal degenerative diseases. However, how to optimize the survival and engraftment of hESC-RPE cells is a great challenge.

**Methods:**

Here, we report hESC-RPE cells that are embedded with polyelectrolytes gelatin and alginate by layer-by-layer (LbL) self-assembly technique, based on the opposite charge of alternate layers. Cells were assessed for cell survival, immunogenicity, and function in vitro and in vivo.

**Results:**

This strategy obviously decreased the immunogenicity of hESC-RPE cells without affecting its activity. LbL-RPE cell transplants into the subretinal space of Royal College of Surgeons (RCS) rats optimized cell engraftment and decreased immunogenicity compared to untreated RPE cell transplants (immunosuppression was not used during the 21-week study). Visual-functional assay with electroretinogram recordings (ERGs) also showed higher B wave amplitudes in RCS rats with LbL-RPE cell transplants.

**Conclusions:**

We demonstrate that transplanted LbL-RPE cells have better viability and grafting efficiency, optimized immunogenicity, and visual function. Therefore, LbL engineering is a promising method to increase the efficacy of hESC-RPE cell transplantation.

## Background

Retinal pigment epithelial (RPE) cells, locating between vessels of the choriocapillaris and light-sensitive outer segments of the photoreceptors, are crucial to the survival and function of photoreceptors [1–3]. Metabolic disorders of RPE cells with age result in photoreceptor death, and this process mainly occurs in the development of age-related macular degeneration (AMD), a commonest cause of blindness in the developed world [3, 4]. Since the RPE has very limited ability to regenerate, a variety of cellular regenerative therapies with a range of RPE cell types are being studied for the treatment of AMD in clinical trial, which include subretinal transplantation of human allogeneic RPE cells, embryonic stem cell-derived RPE (hESC-RPE), or induced pluripotent stem cell-derived RPE (iPSC-RPE) [5, 6]. We and other groups had held greater potential with hESC-RPE transplantation due to relatively convenient access to cells and ethical acceptance [7–10]. Although the retina is generally considered relatively immune-privileged, this allograft transplantation will lead to immune rejection [11]. Immunosuppression should be performed in allogenic RPE cell transplantation for AMD [6], which would be accompanied by serious side effects inevitably, especially for the elder recipients. Strategies to reduce the immunogenicity of RPE cells will facilitate the application of cell therapies.

There are two main approaches to deliver cells into the subretinal space. One is the injection of an RPE cell suspension, and the other involves engrafting a monolayer of RPE cells seeded on a supporting membrane. For the former method, how to ensure that cells are engrafted to the targeted lesion area remains a serious challenge [3, 12, 13]. Recently, we successfully transplanted hESC-RPE suspension [14] into the subretinal space of AMD patients [10]. However, it is difficult to control the cells distribution after surgery.

Layer-by-layer (LbL) self-assembly, a tissue engineering method, has been used to encapsulate living cells thus forming a protective coating that allows continued cell activity and a viable drug delivery system and does not affect cell viability or proliferation at the same time [15–20]. Gelatin and alginate, based on their opposite charges under the same pH conditions, have been used to encapsulate mammalian cells. That is, polycation and polyanion are deposited on the cell surface to form an extracellular coating [17, 19]. Previously, researchers have also tried to develop cell encapsulation with polymers [21] as an approach to reduce activation of host immune responses [22–25]. Alginate-encapsulated cells can function for up to 180 days in vivo, although encapsulation size can affect the immunogenicity of implanted materials [26]. The structure of alginate hydrogels is similar to the extracellular matrix of living tissue. Alginate hydrogels have a wide range of applications in cell transplantation and other research fields. As alginate was purified to a very high purity through a multi-step extraction process, no significant foreign body reaction was caused when it was implanted into animals [21, 27]. Notably, the eye was especially suited to transplantation of LbL-RPE cells as a cell therapy due to its anatomical features and immune privilege [28] to optimize survival and engraftment of the transplanted RPE cells. It has been found that hypoimmunogenic cell not requiring any immunosuppression can be engineered for universal transplantation [29]. Methods to reduce the need for immunosuppression are essential for the broad clinical application of future RPE replacement therapies [30].

Could transplantation of LbL-RPE cells survive without triggering a host immune rejection response and provide a permissive subretinal environment for the maintenance of visual function? We hypothesized that LbL self-assembly with gelatin and alginate was likely to facilitate cell engraftment and inhibit immune response. Although LbL cell encapsulation may represent a suitable source of cells for transplantation therapies, there are still some concerns that need to be addressed. Detailed characterization of immune responses, the engraftment efficiency, and survival rate following transplantation are required if LbL-RPE cells are to be used clinically. To this end, we established RPE cell cultures derived from hESC for LbL coating, then transplanted these cells or uncoated RPE cells into the subretinal space of Royal College of Surgeons (RCS; retinal dystrophic) rats without the use of immunosuppression. We assessed the quality, quantity, and safety of LbL self-assembly by examining cell morphology in vitro, the engraftment efficiency, function, and immunogenicity in vivo. This work suggests further tests on one of the key issues facing RPE transplantation may yield results that can be taken forward for use in clinical trials of retinal degenerative diseases. We hope that the preliminary results of this study can provide new ideas for future RPE replacement therapies.

## Methods

### Preparation and culture of hESC-derived RPE cells

We cultured Q-CTS-hESC-2 cell line [10, 31] using xeno-free Essential 8™ Medium (A1517001, Gibco) to induce differentiation into RPE cells as previously described [10]. Briefly, the differentiation from hESC to RPE cells employs procedures such as super-confluence, acquired pigment foci, and excision. The culture medium for the RPE cells that diffused from the excised pigment foci contained 78% KO-DMEM CTS (Invitrogen), 20% Knock Out SR xenofree CTS (Invitrogen), 1% CTS glutaMAX-1 supplement (Invitrogen), 1% MEM NEAA (Invitrogen), and 1% 2-Mercaptoethanol (Procell). hESC-derived RPE cells were cultured in cell culture dishes at 37 °C in an incubator with 5% CO_2_/95% air, and the medium replaced every 2 days. Proliferating cultures were passaged 1:4, after being digested with CTS™ TrypLE™ Select Enzym (Gibco).

### In vitro RPE and LbL-RPE cell-T cell rejection assay

Purified CD4^+^ T cells (5–8 × 10^5^ cells/well in 96-well plates) from the PBMCs of healthy donors were co-cultured with RPE and LbL-RPE cells (5–8 × 10^3^ cells/well: effector/target ratio = 500:1, and 100:1) for 48 h. T cell activation was evaluated by measuring IFN-γ production using ELISA (Human IFN-γ ELISA Kit II, BD, 550612).

### Flow cytometry

Expression of HLA class I, HLA class II, CD80, CD86, and CD276 by RPE and LbL-RPE cells was examined by FACS analysis. Before staining, cells were incubated with a human Fc block (BD PharMingen, #564765) at 4 °C for 15 min. The cells were stained with the following: anti-HLA class I antibody (PE Mouse anti-Human HLA-A2; BD PharMingen, #558570), anti-HLA class II antibody (FITC Mouse Anti-Human HLA-DR; BD PharMingen, #555811), PE Mouse Anti-Human CD80 (BD PharMingen, #560925), APC Mouse Anti-Human CD86 (BD PharMingen, #560956), and BV421 Mouse Anti-Human CD276 (BD PharMingen, #565829). The following isotype controls were used: mouse IgG2b, κ isotype control, PE—BD PharMingen, #555743; mouse IgG2a, κ isotype control RUO, FITC—BD PharMingen, #555573; mouse IgG1, κ isotype control, PE—BD PharMingen, #555749; mouse IgG1, κ isotype control, APC—BD PharMingen, #555751; and mouse IgG1, κ isotype control, BV421—BD PharMingen, #562438. Cells were incubated at 4 °C for 30 min. RPE and LbL-RPE cells co-cultured with recombinant IFN-γ (100 ng/mL) for 48 h were also prepared. All samples were analyzed on a FACSVerse flow cytometer (BD), and data analyzed with FlowJo software (version 7.6.1).

### Transplantation of RPE and LbL-RPE cells into the subretinal space of RCS rats

RPE and LbL-RPE cells were labeled by CellTracker™ CM-DiI (C7000) before transplantation, which were incubated in 1 μg/mL solution for 5 min at 37 °C, and then for an additional 15 min at 4 °C. RCS rats (21 days old, *n* = 43) were placed under general anesthesia with an intraperitoneal injection of 1% pentobarbital sodium. A small scleral incision between the lateral canthus and 2–3 mm distant from the corneal limbus was made using a 29-gauge needle. We then injected into the subretinal space of the right or left eye 2 μL DPBS containing a 1 × 10^5^ RPE or LbL-RPE cell suspension, or DPBS alone as a sham injection. Rats were sacrificed 5 weeks and 21 weeks after transplantation. For assessment of biostability of the LbL coating of LbL-RPE cells in vivo, RPE cells received 3 layers of LbL coatings, the last layer of gelatin was conjugated with FITC, and were not labeled by CellTracker™ CM-DiI (C7000); then, we injected into the subretinal space of the right eye 2 μL DPBS containing a 1 × 10^5^ gelatin/alginate/gelatin-FITC-encapsulated RPE cell suspension, and rats were sacrificed at 2 weeks, 3 weeks, 5 weeks, and 21 weeks after transplantation. RCS rats were not given any immunosuppression drugs throughout the experiment.

### Statistical analysis

At least three independent experiments were performed for all assays. For statistical analyses, differences between the groups were analyzed using paired *t* test, two-way ANOVA followed by Sidak’s multiple comparisons test, and one-way ANOVA followed by Dunnett’s multiple comparisons test. Values were considered statistically significant if *p* < 0.05. For further details regarding the experimental procedures used in this work, including *Materials and reagents*, *Cell Viability Test with Calcien AM/PI Staining*, *Zeta-Potential Assessment*, *LbL Single-Cell Encapsulation*, *Methyl thiazolyltetrazolium Test*, *Preparation of Fluorescent Reagents in Labeled Gelatin and Alginate*, *Transmission Electron Microscopy (TEM)*, *Scanning Electron Microscopy (SEM)*, *Preparation of photoreceptor rod outer segment*, *Phagocytosis assay*, *Transepithelial electrical resistance (TER) measurements*, *Preparation of Human PBMCs and T Cells*, *Mixed Lymphocyte Reactions with RPE and LbL-RPE cells*, *Animal Experiments*, *Full-field ERG recordings*, *Immunofluorescent Staining*, and *Cell Counting*, see Supplemental Experimental Procedures.

## Results

### Preparation of layer-by-layer encapsulation of RPE cells

**Fig. 1:**
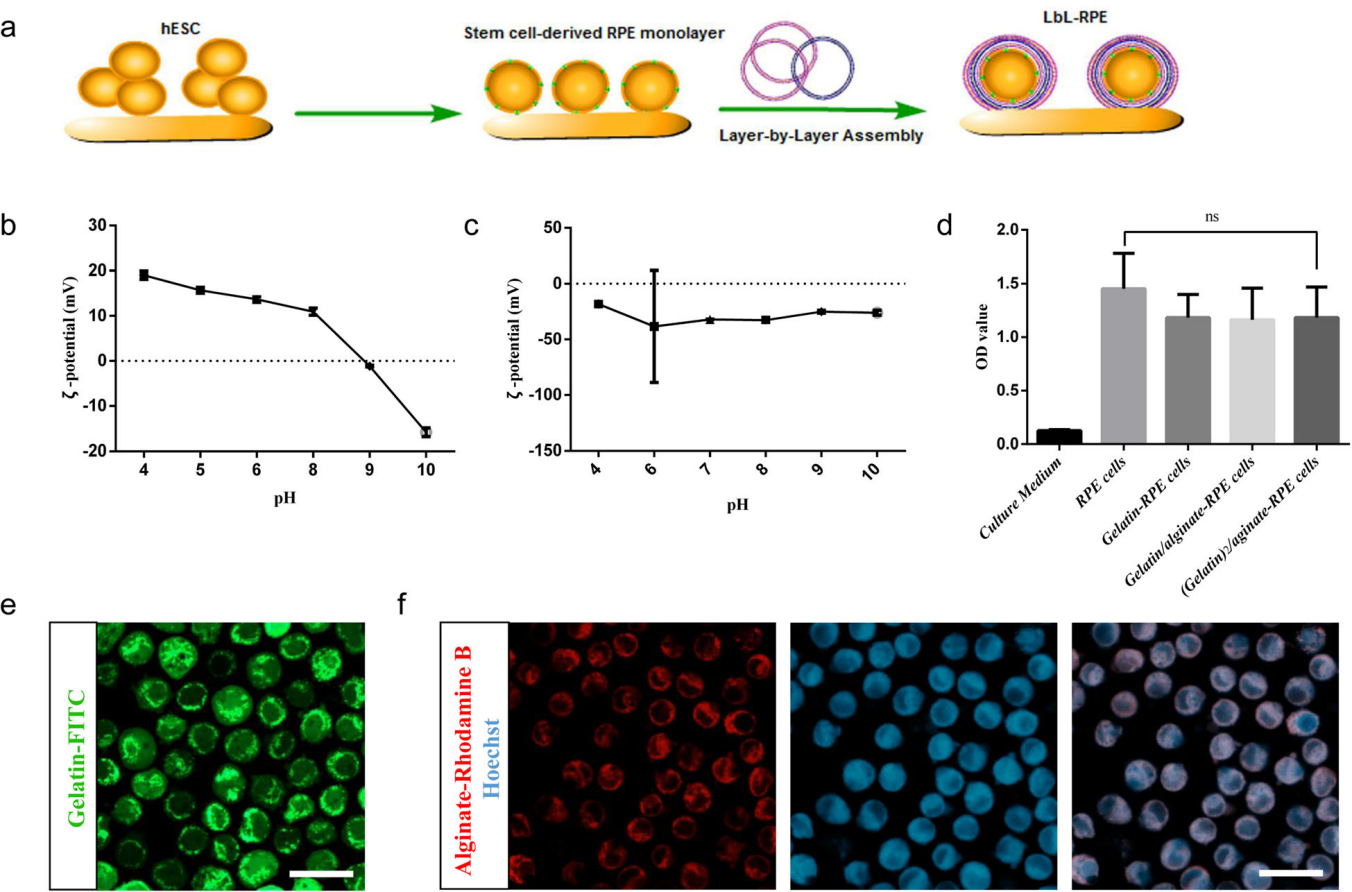
Preparation of layer-by-layer encapsulation of RPE cells. **a** Schematic model of layer-by-layer self-assembly technique. Polycations and polyanions provide alternating layers that coat RPE cells due to the interaction of opposite charges. **b** Surface charge of gelatin-type A as a function of pH; the isoelectric point is approaching 9. **c** Surface charge of alginate as a function of pH; the isoelectric point is approaching 4. **d** MTT assay showed cell viability of untreated and LbL-RPE cells when cultured for 3 days. Data represent the mean ± SEM of three independent experiments. There were no significant differences (*p* > 0.05) between untreated RPE and LbL-RPE cells. **e** Gelatin/alginate/gelatin-FITC-encapsulated RPE cells; the last layer of gelatin was conjugated to FITC for detection using fluorescence microscopy. Scale bars 10 μm. **f** Gelatin/alginate-Rhodamine B-encapsulated RPE cells; the last layer of alginate was conjugated to Rhodamine B. Nuclei were stained with Hoechst (blue). Scale bars 10 μm

The differentiation of hESC into RPE cells was summarized in Figure S1A, which includes three steps: super-confluence, acquired pigment foci, and excision. Thus, we observed clusters of pigmented RPE cell monolayers that exhibited their unique cobblestone morphology at the edges of clusters. RPE cells were encapsulated as schematically shown in Fig. 1a. The principle model of LbL self-assembly is that the polycations and polyanions attach to each other in a sequential layer-by-layer way due to the interaction of opposite charges. The sequential application of the ions thus provides multiple layers on RPE cell surface. To assess the isoelectric point (IEP) of gelatin and alginate, we measured the zeta-potential and obtained the surface charge of gelatin-type A and alginate as a function of pH. The IEP of gelatin is approaching 9, and that of alginate is approaching 4. Thus, they display opposite charges at physiological pH, which is between 6 and 8 (Fig. 1b, c). We then examined the cytotoxicity of LbL encapsulation with 3 layers of materials, that is (gelatin)_2_/alginate. Figure 1d shows that the LbL-RPE and untreated RPE cells cultured for 3 days had similar MTT absorbance, suggesting that the LbL encapsulation did not significantly influence RPE cell viability. Calcien AM/PI staining also showed the viability of LbL-RPE cells (~ 95.6%), further strengthening the thesis that gelatin and alginate are non-toxic (Figure S1B). Gelatin and alginate conjugated to fluorescent probes (FITC and Rhodamine B, respectively) were used to confirm the LbL cell coating (Fig. 1e, f).

### Effects and characterization of layer-by-layer assembly coated on RPE cells

**Fig. 2:**
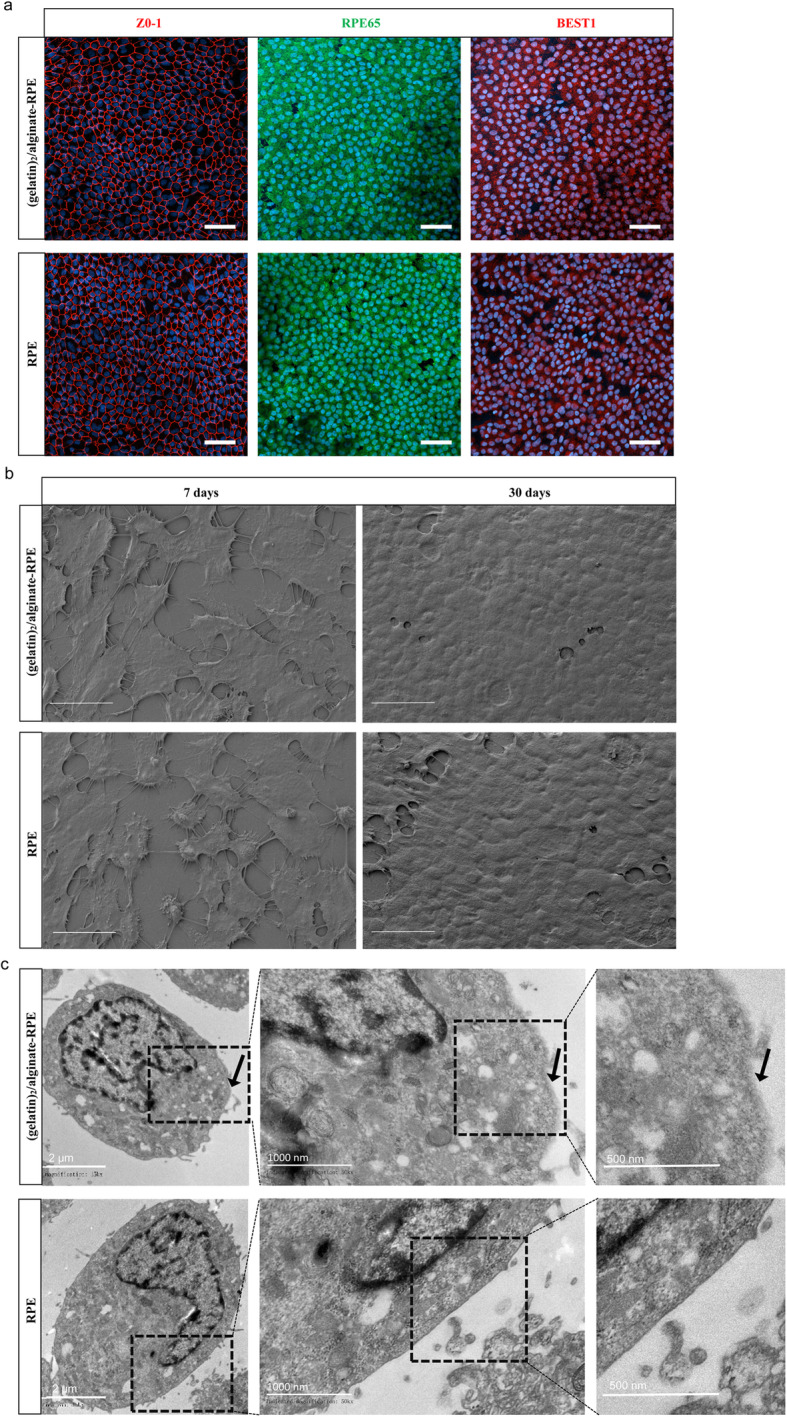
Effects and characterization of layer-by-layer assembly coated on RPE cells. **a** Immunostaining for typical RPE markers (ZO-1, RPE65, and BEST1) in (gelatin)_2_/alginate-encapsulated RPE and untreated RPE cells. Scale bar 20 μm. **b** SEM images of untreated RPE cells, and (gelatin)_2_/alginate-encapsulated RPE cells after 7 days and 30 days in culture. Scale bar 10 μm. **c** Different magnifications of TEM images of (gelatin)_2_/alginate-encapsulated RPE cells and untreated RPE cells. Arrows indicate the materials on the cell surface

Both RPE and LbL-RPE cultured cells expressed typical RPE markers (ZO-1, a tight junction marker; RPE65, the retinoid cycle-related marker; and BEST1, chloride channel-related marker) on immunocytochemistry (Fig. 2a). Scanning electron microscope (SEM) was then used to examine the surface morphology of RPE and LbL-RPE cells. Both RPE and LbL-RPE cells exhibited numerous cell contacts after 7 days and 30 days in culture (Fig. 2b). The LbL coating on RPE cells was further confirmed using transmission electron microscope (TEM), which showed that the gelatin/alginate layers on the cell surface were 4~8 nm thick (calculated by ImageJ) (Fig. 2c). LbL coating formed a thin-film extracellular coating by depositing polycations and polyanions onto cells.

### Morphological and functional assessment of a LbL coating on RPE cells

**Fig. 3:**
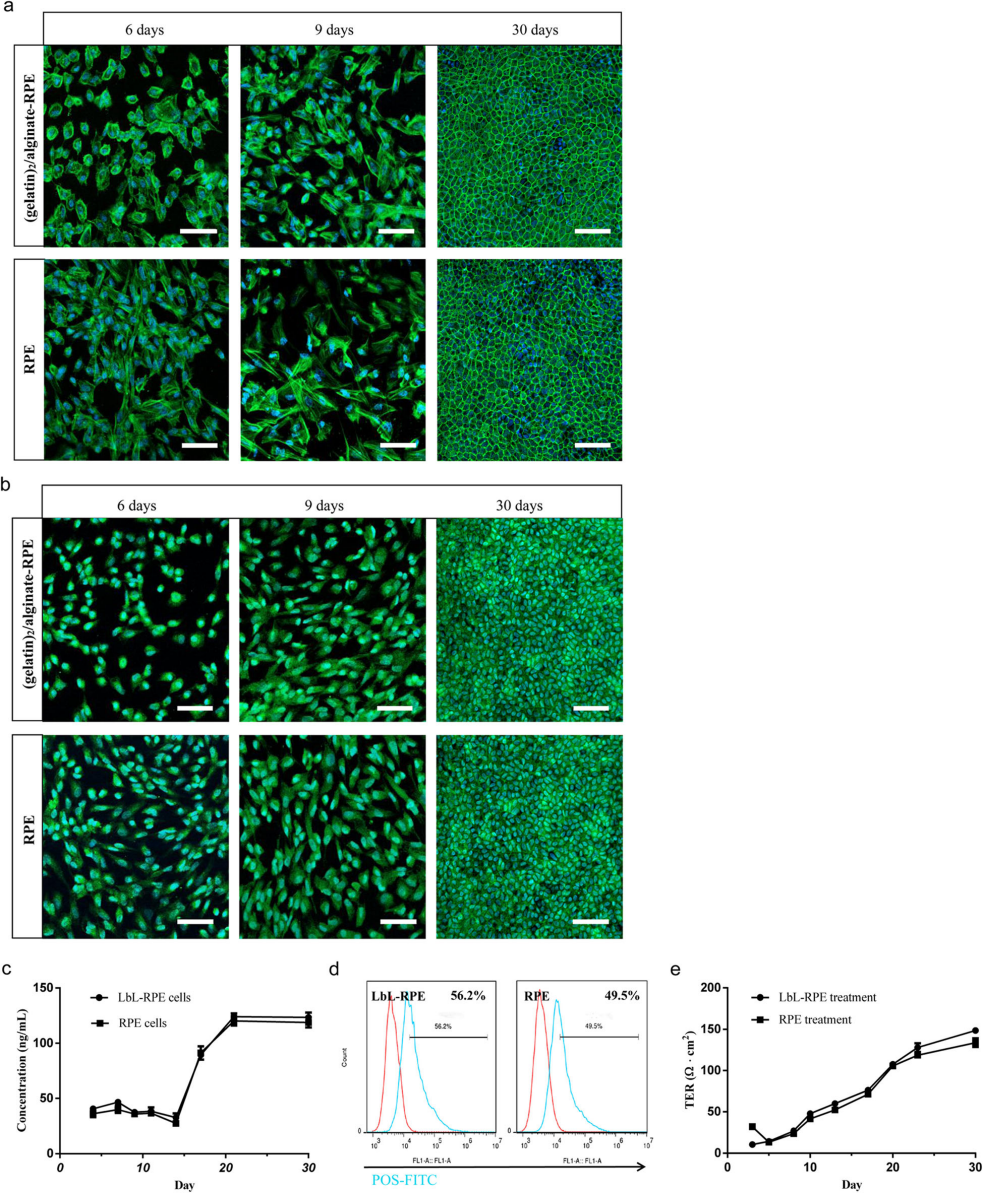
Morphological and functional assessment of untreated RPE and LbL-RPE cells in vitro*.* **a** Differences in F-actin (green) morphology in cultures of untreated RPE and LbL-RPE cells stained with phalloidin indicate there were no differences between untreated and treated cells on days 6, 9, and 30 (cell nuclei—blue Hoechst stain). Scale bar 20 μm. **b** Immunostaining for PEDF (green) in untreated RPE and LbL-RPE cells was used as a marker to demonstrate continued presence of PEDF on days 6, 9, and 30 (cell nuclei—blue Hoechst stain). Scale bar 20 μm. **c** PEDF release curves for untreated RPE and LbL-RPE cells. Cells were cultured at pH 6.5–7.45 in 12-well plates. PEDF concentration in the medium was tested with an ELISA kit. Data represent the mean ± SEM of three independent experiments (4 to 30 days in culture; *p* > 0.05). **d** Measurement of ability to phagocytose photoreceptor outer segments (POS) by RPE and LbL-RPE cells. RPE and LbL-RPE cells were cultured with FITC-POS at 37 °C for 3 h and analyzed by flow cytometry. RPE cells cultured without POS were used as controls (shown in red). **e** Transepithelial electrical resistance (TER) measurements of untreated and LbL-RPE cells cultured for 3 to 30 days were used to characterize epithelial barrier function (mean ± SEM; *n* = 6; *p* > 0.05)

F-actin staining as shown by phalloidin staining (Fig. 3a) on days 6, 9, and 30 showed that there were no apparent differences on the growth of the RPE and LbL-RPE cells after 9 days in culture. RPE and LbL-RPE cultured cells were assessed for growth states and were observed and photographed under microscopy on days 4, 7, 9, 18, 21, and 30 (Figure S1C and S1D). Phase contrast images of LbL-RPE and untreated RPE cells were shown at 21 and 30 days after differentiation. Both RPE and LbL-RPE cells became confluent on a culture dish which displayed some features typical of RPE cells after 21 days in culture, for example, RPE and LbL-RPE cells contained a typical characteristic RPE cobblestone appearance (Figure S1D). Pigment epithelium-derived factor (PEDF) has neurotrophic functions and is an anti-angiogenic protein specifically expressed on the cell surface of normal RPE cells [32]. Anti-PEDF was used as a cell surface marker to determine the normal function of RPE and LbL-RPE cells after 6, 9, and 30 days in culture. PEDF staining on LbL-RPE and untreated RPE cells was similar, suggesting that LbL process did not significantly affect the normal deposition of PEDF (Fig. 3b). PEDF release from RPE and LbL-RPE cells was also measured using ELISA from day 2 to 30. Results showed that the amount of PEDF secreted by RPE and LbL-RPE cells was not significantly different (Fig. 3c). Similarly, the phagocytic function of LbL-RPE and RPE cells, demonstrated by their ability to phagocytize photoreceptor outer segments (POS) and analyzed by flow cytometer (Fig. 3d), showed that there were no significant differences (LbL-RPE, 56.2%; RPE cells, 49.5%). To demonstrate the presence of tight junctions between RPE cells, we performed functional transepithelial electrical resistance (TER) measurements. TER measurements showed a time-dependent increase in LbL-RPE and RPE cell cultures between 3 and 30 days in culture when grown on Transwell membranes; TER after 30 days reached 150.25 ± 6.8 and 131.25 ± 16.8 (Ω cm^2^), respectively (*p* > 0.05; Fig. 3e).

### Immunogenicity of RPE and LbL-RPE cells in vitro

**Fig. 4:**
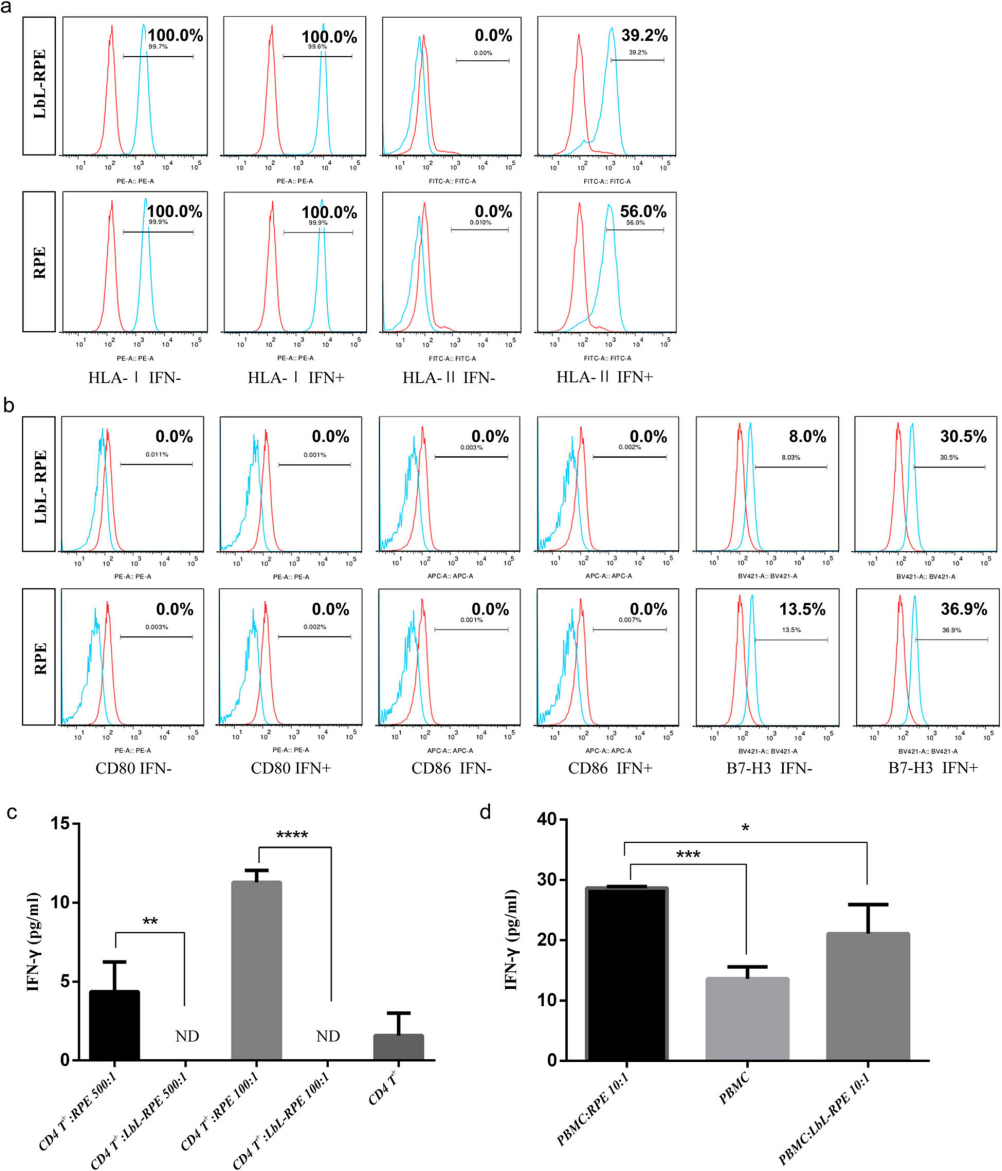
Immunogenicity of RPE and LbL-RPE cells in vitro. **a** Expression of HLA class I and HLA class II on RPE and LbL-RPE cells. RPE and LbL-RPE cells in the presence of recombinant IFN-γ (100 ng/mL) were cultured for 48 h then stained with anti-HLA-I or HLA-II antibodies. The isotype control is shown in red. Numbers in the histogram indicate that the percentage of positive cells was less than 0.5% when determined by isotype-specific controls. **b** Detection of co-stimulatory molecules using anti-CD80, CD86, or B7-H3 (blue) in RPE and LbL-RPE cell cultures with or without IFN-γ. Isotype control is shown in red. Numbers in the histogram indicate that the percentage of positive cells was less than 0.5% when determined by isotype-specific controls. **c** Production of IFN-γ in CD4^+^ T cells exposed to RPE or LbL-RPE cells. Purified CD4^+^ T cells (5 × 10^5^, from human PBMCs) were co-cultured with RPE or LbL-RPE cells for 48 h, and the levels of IFN-γ in the supernatants measured (data represent the mean ± SEM of three independent experiments; ND, not detected; ****p < 0.0001; **p < 0.01). **d** Detection of inflammatory cytokine IFN-γ by human PBMCs when co-cultured with RPE or LbL-RPE cells. PBMCs (5 × 10^5^) were cultured with RPE or LbL-RPE cells for 120 h, and the levels of IFN-γ in the supernatants measured (data represent the mean ± SEM of three independent experiments; ***p < 0.001; *p < 0.05)

We first examined the in vitro expression of MHC proteins on the cell surface, as these play an important role in mediating immune responses. RPE and LbL-RPE cells constitutively expressed HLA class I, but not class II. The ability of interferon-gamma (IFN-γ) to regulate the in vitro surface expression of MHC proteins by RPE and LbL-RPE cells was used to examine the potential effects of any IFN-γ-mediated inflammation induced by transplanting the cells [33]. LbL-RPE cells and RPE cells were co-cultured with recombinant IFN-γ (100 ng/mL) for 48 h. In response to IFN-γ pretreatment, LbL-RPE cells began to express HLA class II (39.2%). Similarly, RPE cells also began to express HLA class II but to a greater degree (56.0%) (Fig. 4a). We next examined the expression of CD80 (B7-1), CD86 (B7-2), and CD276 (B7-H3) co-stimulatory molecules. RPE and LbL-RPE cells did not express CD80 (B7-1) or CD86 (B7-2) when cultured with or without IFN-γ pretreatment, but constitutively expressed CD276 (B7-H3) co-stimulatory molecules. There was an increase in the number of LbL-RPE cells expressing CD276 (B7-H3) after the addition of IFN-γ (8% vs. 30.5%), and a similar increase in the number of RPE cells expressing CD276 (B7-H3) was also seen (13.5% vs. 36.9%) (Fig. 4b). We then examined whether RPE or LbL-RPE cells could be directly recognized by T cells. We used purified CD4^+^ T cells (88%) cultured with RPE or LbL-RPE cells in the presence of recombinant IL-2 (CD4^+^ T-RPE cell cytokine assay) and collected the supernatants after 48 h to measure IFN-γ [34]. The ratio of T cells to RPE or LbL-RPE cells in the cultures was 5 × 10^5^ T cells and 5 × 10^3^ (100:1) or 1 × 10^3^ (500:1) RPE or LbL-RPE cells. T cells produced significant amounts of IFN-γ in proportion to the number of RPE cells, but the T cells did not respond to the LbL-RPE cells and failed to express IFN-γ (Fig. 4c). Thus, it is apparent that LbL-RPE cells are poorly immunogenic compared to untreated RPE cells in culture. A mixed lymphocyte reaction (MLR) assay of fresh peripheral blood mononuclear cells (PBMCs) mixed with RPE or LbL-RPE cells, in the presence of interleukin-2 (IL-2), was used to examine IFN-γ production and to judge the effect of the immune response (effector/target ratio = 10:1). Supernatants collected for 96–120 h from PBMC-RPE cell cultures contained high levels of IFN-γ compared with supernatants from PBMC-LbL-RPE cultures (*p < 0.05) and further confirmed the lower immunogenicity of LbL-RPE cells in culture (Fig. 4d).

### Biostability of the LbL coating, survival, and functional assessment of RPE and LbL-RPE cells in vivo

**Fig. 5:**
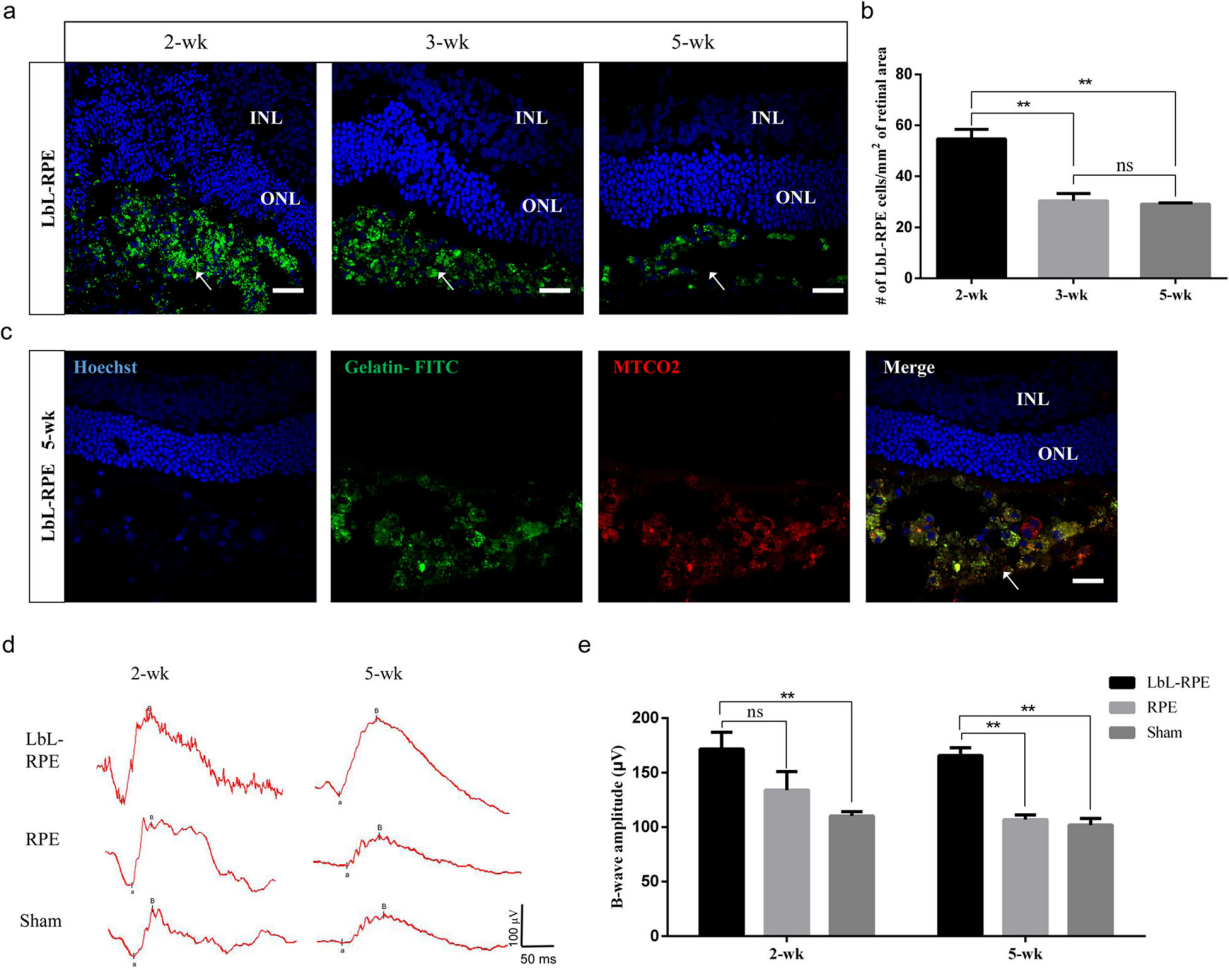
Biostability of the LbL coating and functional assessment of RPE and LbL-RPE cells from 2 to 5 weeks after transplantation. **a** Images of LbL-RPE cells after transplantation in RCS rats from 2 to 5 weeks, showing gelatin/alginate/gelatin-FITC-encapsulated RPE cells could effectively engraft around the grafted area. RPE cells received 3 layers of LbL coating; the last layer of gelatin was conjugated with FITC. Scale bar 20 μm. **b** The number of gelatin/alginate/gelatin-FITC-encapsulated RPE cells around the transplanted area at 2, 3, and 5 weeks after transplantation (bars: mean ± SEM; *n* = 3; **p < 0.01). **c** Immunofluorescence staining observed by confocal microscopy showed colocalization of human mitochondria and Hoechst in gelatin/alginate/gelatin-FITC-encapsulated RPE cells at 5 weeks after transplantation. Scale bar 20 μm. **d** Representative average scotopic ERG traces obtained from RCS rats at 2 and 5 weeks after transplantation (*n* = 8 rats per condition). **e** The average B wave amplitude is well maintained in animals with LbL-RPE cell grafts compared to grafts containing untreated RPE cells and suggests some maintenance of visual function as a result (mean ± SEM.; **p < 0.01)

**Fig. 6:**
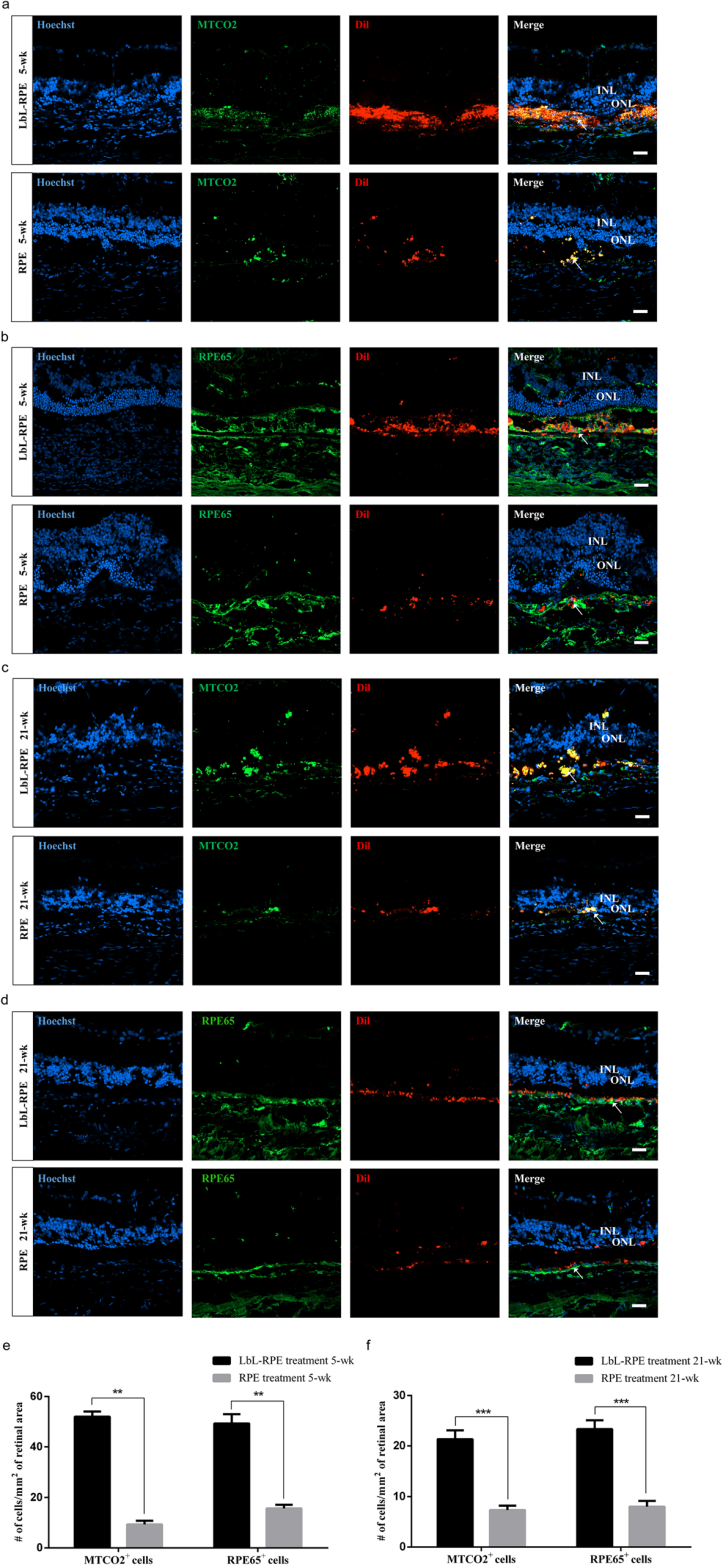
Survival assessment of RPE and LbL-RPE cells in vivo*.* **a**–**d** Immunofluorescence staining observed by confocal microscopy. Transplanted cells (pre-labeled with Dil (red)) expressed human mitochondria (green) or RPE65 (green) markers. Transplanted LbL-RPE cells remained at the injection site at 5 and 21 weeks after surgery (arrowhead). Only a limited number of untreated RPE cells remained in graft area at 5 and 21 weeks after surgery (arrowhead). Arrows indicated viable transplanted RPE and LbL-RPE cells which were human mitochondria or RPE65, Hoechst and Dil positive. Scale bar 20 μm. **e**, **f** Quantitation showing the number of cells labeled with the human mitochondrial or RPE65 antibody at 5 and 21 weeks after RPE and LbL-RPE cell transplants (mean ± SEM; ***p < 0.001; **p < 0.01; *n* = 8)

We transplanted hESC-derived RPE and LbL-RPE cell suspensions into the subretinal space of 3-week-old RCS rats [35–37] to assess the biostability of the LbL coating (degradation of LbL layers) and functionality of the retina. Figure 5a shows efficiently engrafted LbL-RPE cells pre-labeled with gelatin/alginate/gelatin-FITC (3 layers, the last gelatin layer conjugated to FITC) could be observed around the grafted area from 2 to 5 weeks. These results show that the LbL layers were still present on the cell surface up to 5 weeks after transplantation, albeit the number of labeled cells around transplanted site was significantly less than that seen after 2 weeks (**p < 0.01; Fig. 5b). We then confirmed the survival of LbL-RPE cells at 5 weeks using gelatin/alginate/gelatin-FITC pre-labeling and immunostaining for a human-specific marker, i.e., antibodies against the human mitochondria in the LbL-RPE cells. Double-labeled cells were considered to be surviving transplanted LbL-RPE cells (Fig. 5c). Additionally, the fluorescence faded away into the transplanted area after 21 weeks, indicating that LbL layers had been degraded in vivo. Our functional analysis included electroretinograms (ERGs), more specifically the B wave amplitude, recorded 2 and 5 weeks after transplantation. The B wave amplitudes in animals with LbL-RPE-treated transplants at 2 weeks were significantly higher than that of the sham group, but not significantly different compared to animals receiving untreated RPE cells. B wave amplitudes at 5 weeks after LbL-RPE cell transplants were significantly higher than those recorded after untreated RPE cell transplants or sham injections (Fig. 5d, e). We also performed functional analysis with ERGs for 21 weeks, but both groups showed unrecorded wave amplitudes, which may be due to the disease process in the most advanced stage of RCS rats (data not shown). We also assessed the survival of RPE or LbL-RPE cells at 5 weeks and 21 weeks after transplantation using Dil pre-labeling and immunostaining for the human mitochondria or RPE65 (Fig. 6a–d and Figure S2A-D). Double-labeled cells were considered to be transplanted RPE or LbL-RPE cells. The results show that the number of surviving transplanted LbL-RPE cells at 5 weeks and 21 weeks (mean: MTCO2^+^ cells, ~ 52 cells/mm^2^/~ 21 cells/mm^2^; RPE65^+^ cells, ~ 49 cells/mm^2^/~ 23 cells/mm^2^) was significantly higher than that of untreated RPE cells (mean: MTCO2^+^ cells, ~ 9 cells/mm^2^/~ 7 cells/mm^2^; RPE65^+^ cells, ~ 16 cells/mm^2^/~ 8 cells/mm^2^) (Fig. 6e, f). LbL-RPE cell transplants also optimized cell engraftment compared to untreated RPE cell transplants. The transplanted LbL-RPE cells had less diffusion. Cell survival and engraftment efficiency were enhanced if LbL-RPE cells were used.

### Immunogenicity of RPE cells or LbL-RPE cells in vivo

**Fig. 7:**
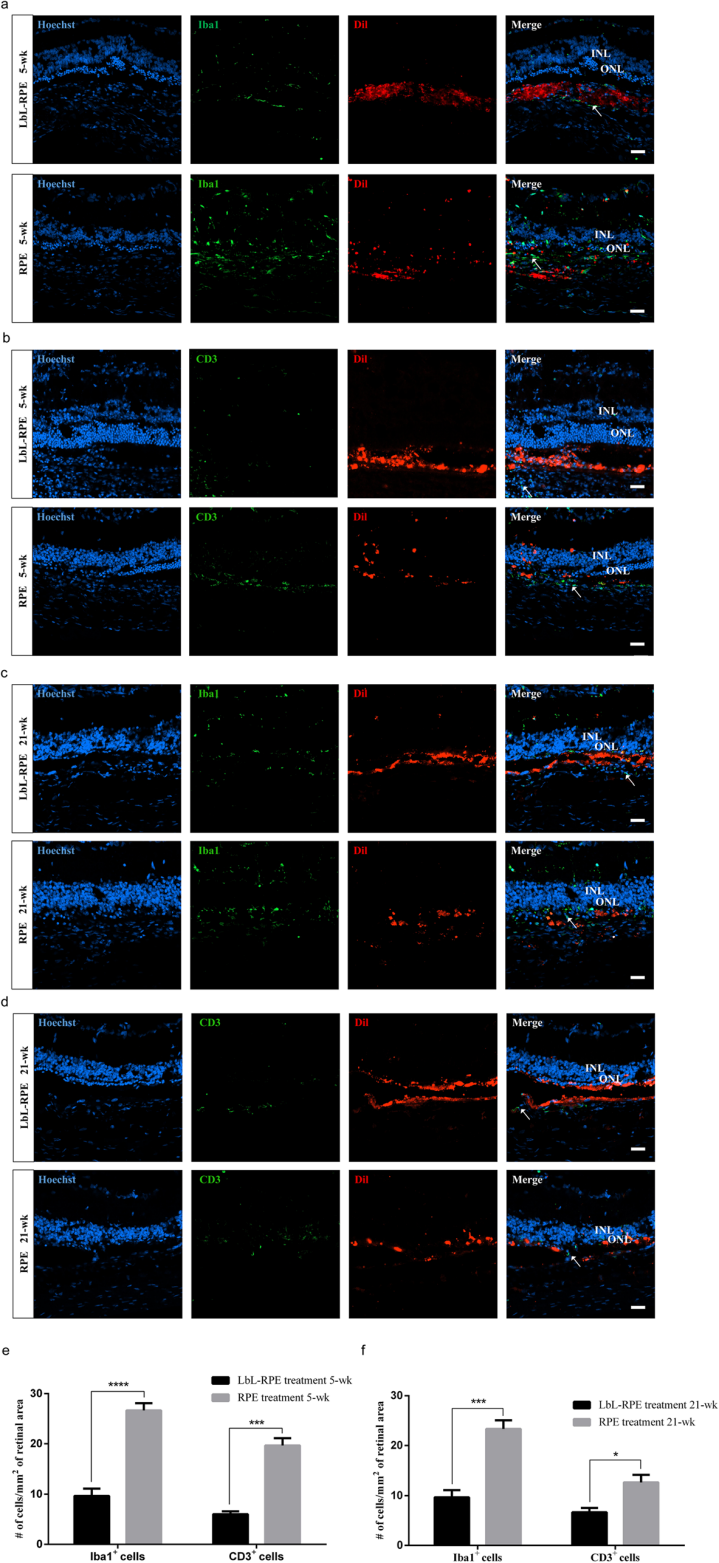
Immunogenicity of RPE cells or LbL-RPE cells at 5 and 21 weeks after transplantation. **a**–**d** Photomicrographs showed the labeling of RCS rats’ retinal sections at 5 and 21 weeks after transplantation. Anti-Iba1/CD3 antibody (green); many Iba1^+^ cells (arrow) invaded the INL/ONL after RPE transplants, but were poorly labeled after LbL-RPE transplants (RPE and LbL-RPE cells were pre-labeled with Dil (red)). There were numerous CD3^+^ cells (arrow) which infiltrated in the RPE retinas. CD3^+^ cells were very sparse in LbL-RPE transplants. Scale bars 20 μm. **e**, **f** The number of positive Iba1^+^ and CD3^+^ cells around the transplanted retinas at 5 and 21 weeks after transplantation (mean ± SEM; ****p < 0.0001; ***p < 0.001; *p < 0.05; *n* = 8)

Retinal sections were examined for inflammatory cell activity after RPE and LbL-RPE transplants at 5 weeks and 21 weeks after transplantation. Frozen RCS rat retinal sections were stained with anti-ionized calcium-binding adapter molecule 1 (Iba1) and CD3 antibodies. In response to RPE transplants, Iba1^+^ cells (microglia) invaded the inner and outer nuclear layers (INL and ONL, respectively), and abundant CD3^+^ had infiltrated the retina. In addition, we also observed that the survival rate of RPE cells in the subretinal space was less than that of LbL-RPE cells. In contrast, transplanted LbL-RPE cells survived in the subretinal space and the retinal sections were poorly infiltrated with Iba1^+^ and CD3^+^ cells around the transplant site (Fig. 7a–d, Figure S3A-B and S3D-E). Following sham injections at 5 weeks after transplantation, we observed Iba1^+^ cells in the retina but not CD3^+^ cells (Figure S3C) and this was similar to the finding in normal RCS rats (data not shown). Quantitative analysis showed that the number of positively labeled immunogenic inflammatory cells around the transplant of the RPE cell transplant group at 5 weeks and 21 weeks (mean: Iba1^+^ cells, ~ 27 cells/mm^2^/~ 23 cells/mm^2^; CD3^+^ cells, ~ 20 cells/mm^2^/~ 13 cells/mm^2^) was significantly greater than that seen following LbL-RPE cell transplants (mean: Iba1^+^ cells, ~ 10 cells/mm^2^/~ 10 cells/mm^2^; CD3^+^ cells, ~ 6 cells/mm^2^/~ 7 cells/mm^2^) (Fig. 7e, f). The results indicate that LbL encapsulation decreased the immunogenicity of the RPE cell transplants in vivo.

## Discussion

In this study, we report the production of hESC-RPE cells for LbL coating. These LbL-RPE cells exhibit the morphological characteristics, gene expression, and in vitro and in vivo function of hESC-RPE cells. The biosafety, grafting efficiency, and visual-functional improvement of transplanted LbL-RPE cells in vivo were confirmed, which indicated that LbL provides a protective microenvironment for the survival and engraftment of LbL-RPE cells in the subretinal space. It can be seen that tissue engineering technology may have more potential therapeutic and future clinical application value. RPE cells play a critical role in retinal degenerative diseases, and transplantation of RPE cells has been trialed as a promising curative approach [8, 38]. Considerable attention has been paid to hESC as a source of RPE cells [3, 9, 10]. There are no reports in the literature that RPE cells form tumors, suggesting that hESC-derived RPE cells will not result in such adverse outcomes. In patients with retinal degeneration disease, the RPE and Bruch’s membrane are impaired in the lesion site, and transplanted RPE cells need a collagen-rich extracellular matrix to engraft [33]. LbL is similar to Bruch’s membrane-like biomimetic scaffold that can facilitate the survival and promote the functions of transplanted LbL-RPE cells in vivo. LbL self-assembly optimizes the chance of the cell attachment to Bruch’s membrane, so as to maximize the potential of the transplanted cells to integrate with the native RPE, and then rescue compromised perimacular tissue.

Gelatin and alginate are both natural biocompatible polymers and ideal polyelectrolytes for LbL encapsulation. Gelatin comes from collagen [17]. Alginate is composed of β-d-mannuronate and α-l-guluronate through a 1–4 glycosidic linkage with high affinity for divalent ions [39, 40]. They are gradually transformed into a soluble form by enzymatic or nonenzymatic reactions in vivo. Studies of LbL cell encapsulation by gelatin and alginate and their subsequent transplantation have been shown to have some degree of efficacy [17, 19]. Since our transplanted cells grew in the subretinal space, a potential cavity between neural retinal and RPE layer, the coating materials would not invade into the retinal vessels due to the blood-retinal barrier [41]. And their degradation products had no toxic to the retina. So the retinal complication, such as retinal vessel occlusion, would not occur after LbL-RPE cell transplantation. LbL cells have been used in cell therapy and drug delivery systems based on good LbL biocompatibility and the sustained release of pharmaceutically active drugs to treat diseases [19]. Cell encapsulation can be capable of overcoming the need for immunosuppression by shielding the encapsulated cells from the host immune system [42]. Encapsulation of single RPE cells using tissue engineering techniques created a defined microenvironment for RPE cells. How to avoid transplant rejection by the host and to facilitate transplant engraftment and survival are the focus of this study. On the basis of the above, we used RPE cells derived from hESC, and subsequently encapsulated with gelatin and alginate in a LbL manner to verify its potential application in the treatment of retinal degenerative diseases.

The isoelectric point of gelatin (type A) is approaching 9, being similar to the native extracellular matrix. Alginate is widely used in tissue engineering [17, 19, 21], and the isoelectric point of alginate is approaching 4. The LbL-RPE cells formed monolayers in culture and maintained their outer membrane. In agreement with other reports [17, 19], a LbL coating using gelatin/alginate did not affect cell viability, and cells were able to survive and proliferate in vitro. LbL-RPE cells showed typical RPE behavior, lost pigment cobblestone-like morphology in the process of proliferation, and formed a monolayer of polygonal cuboidal pigmented epithelium once confluent [43]. The thickness of LbL layer was calculated to be 4~8 nm in agreement with previous reports [17, 44]. In our study, LbL-RPE cells could form polarized monolayer with tight functional junction in vitro. The persistence of LbL nanocoating was also demonstrated by using a fluorescent microscope. Our previous study showed the coating materials would last for about 10 days in culture dish [19]. Additionally, we confirmed that LbL layers could be maintained after 5 weeks of subretinal transplantation and almost completely degraded at 21 weeks. A LbL coating of gelatin/alginate facilitated RPE cell engraftment, prolonged cell survival, and helped to maintain cell function in vivo.

Immune rejection after RPE cell transplantation is usually elicited by a T cell-mediated immune response [34]. Both T lymphocytes and inflammatory cytokines play an important role in immune rejection, inducing antigen recognition, and secretion of interferon-γ [34, 45–47]. Cytokine interferon-γ (IFN-γ) [34, 47] is important in transplant rejection as the inflammatory cytokine leads to major histocompatibility complex (MHC)-I and MHC-II expression on RPE cells. T cell activation and a MLR assay are the tools of choice when evaluating immune rejection responses in vitro [33, 34]. RPE cells can induce immune responses in culture as shown by immunofluorescent staining, the MLRs, and the T cell activation assay. The LbL coating reduced the immunogenicity of RPE cells. We have confirmed that RPE and LbL-RPE cells express HLA-I but not HLA-II if IFN-γ is not used as a pretreatment. Analysis of RPE and LbL-RPE cells showed that, except for B7-H3, co-stimulatory molecule expression for CD80 (B7-1)/CD86 (B7-2) was undetectable. IFN-γ upregulated RPE and LbL-RPE cell HLA-II and B7-H3 co-stimulatory molecule expression, but this was lower in LbL-RPE cell cultures. In the T cell activation assay, supernatants from CD4^+^ T cell-RPE cell cultures contained high levels of IFN-γ compared with that of CD4^+^ T-LbL-RPE cell cultures. In a mixed lymphocyte reaction, supernatants from peripheral blood mononuclear cell^+^-RPE cell cultures also contained high levels of IFN-γ compared with that of LbL-RPE cell cultures. These culture results suggest that infiltrating T cells have a limited capacity for antigen processing and presentation in response to LbL-RPE cells. When immunosuppressive medication was not paired with the transplant, the tissues around the RPE cells were invaded by numerous inflammatory Iba1^+^ or CD3^+^ cells and the transplanted RPE cells did not survive well in the subretinal space. In contrast, higher numbers of transplanted LbL-RPE cells survived, better engraftment, and the region was poorly invaded by Iba1^+^ microglia/macrophages, or CD3^+^ T cells. The mechanism of reduced immune rejection induced by LbL-RPE cells is not clear. We speculate that the mechanism may be as follows: First, the decreased permeability of LbL-RPE cells causes the inhibition of immune molecular recognition. The other is that the chemical modification of gelatin-alginate-gelatin affects the recognition of HLA-II by immune cells. Third, the basic characteristics of RPE cells changed after encapsulation, such as the downregulation of HLA-II on cell surface leading to immune escape. There are complex inflammatory stimulating factors such as IFN-γ and TNF-α in the local microenvironment of the diseased retina. Can LbL-RPE cells reduce its immune response by inhibiting the function of IFN-γ, or TNF-α receptor? Therefore, the function of IFN-γ or TNF-α receptor in the LbL-RPE cells stimulated by inflammatory factors deserves further study. We speculate that the inflammatory factors IFN-γ or TNF-α stimulate the expression of HLA-II in the microenvironment of pathological retina. LbL-RPE cells may effectively inhibit the expression of HLA-II through the IFN-γ or TNF-α receptor pathway, so as to prolong the survival of transplanted cells and improve the transplantation effect.

We confirmed that the grafted RPE and LbL-RPE cells were positively labeled with anti-MTCO2 or RPE65 for at least 21 weeks after transplantation in RCS rats without exogenous immunosuppression. LbL-RPE cells consisted of pigmented cells that secreted PEDF and displayed phagocytosis of POS in vitro, an activity which is similar to that of native RPE cells [3]. The TER results suggested that both LbL-RPE and untreated RPE cells possess tight junctions and epithelial barrier function in culture, and accord well with results obtained from native RPE cells (206 ± 151 (Ω cm^2^)) [48]. Retinal ERG recordings in RCS rats further demonstrated that some residual vision function was maintained for up to 5 weeks after LbL-RPE cell transplants compared to non-treated transplants. LbL can enhance the response to light stimuli in vivo and maintain the visual-electrophysiological function in transplanted area. Transplantation of LbL-RPE cells into the subretinal space of RCS rats is safe and effective in promoting cell survival and function.

Our protocol has the advantage that the process requires only a relatively small-scale culture and performance system. LbL encapsulation of RPE cells using our protocol can generate enough cells to cover the macula area. LbL self-assembly was allowed for safe and effective administration of LbL-RPE cells to the target sites in the retina by its self-assembled thin films. Several groups have also tried to improve graft survival and visual function by transplantation of polarized monolayers or intact sheets of RPE cells [33, 49, 50]. However, RPE cell-sheet transplantation requires a complicated, invasive surgical procedure [33]. LbL self-assembly is an injectable device and cell encapsulation for RPE cell at single-cell level; it is attractive due to its innovative self-assembly, minimal-invasive mode, and simplicity. LbL-RPE cells could offer advantages over RPE suspensions and RPE cell sheets. Additionally, various cell factors can be co-loaded into LbL assembly to provide additional benefits, such as a supply of supplemental protein factors that promote survival and maintenance of the fully functional transplanted cells [17, 19]. Self-assembly outer layer provided a protective film to protect LbL-RPE cells from host immune rejection, optimized the engraftment efficiency of RPE cells, and maintained cell survival and functionality. Our findings provide a new idea for the treatment of retinal degeneration diseases by subretinal implantation of LbL-RPE cells.

## Conclusions

We demonstrated that LbL could optimize the adhesion, survival, and function of RPE cells. LbL self-assembly at single-cell level can facilitate RPE cells’ better engraftment, less diffusion, and low immunogenicity, thus making the immune and inflammatory microenvironment more amenable to RPE transplants. Retinas receiving in vivo LbL-RPE cell transplants exhibited retention of ERG amplitudes and engrafted successfully, as well as showing the safety and viability of the RPE cells. The results demonstrated the potential application of cells encapsulated by LbL transplantation for rescuing macular degeneration.

## Supplementary Information

**Additional file 1: Figure S1.** Generation of hESC-RPE cells and effects of layer-by-layer assembly coated on RPE cells (A) A schematic overview of differentiation of hESC into RPE cells. The differentiation from hESC to RPE cells requires three steps: super-confluence, acquired pigment foci, and excision. RPE cells are the cells that diffuse from the excised pigment foci. (B) Calcien AM/PI (propidium iodide) assay showed cell viability of LbL-RPE cells when treated with (gelatin)_2_/alginate. Lower magnification image was observed by fluorescence microscope. Calcien AM (green) showed viable cells, while PI (red) showed dead cells. (Cell viability = (Total cells - number of dead cells)/Total cells × 100%; cell viability was ~ 95.6%). Scale bar: 200 μm. (C) Growth states of LbL-RPE and untreated RPE cells on days 4, 7, 9, and 18. Scale bar: 200 μm. (D) Phase contrast images of LbL-RPE and untreated RPE cells at 21 and 30 days after differentiation. Scale bar: 100 μm. **Figure S2.** Survival assessment of RPE and LbL-RPE cells in vivo (A-D) Immunofluorescence staining observed by confocal microscopy. Transplanted cells (pre-labeled with Dil (red)) expressed human mitochondria (green) or RPE65 (green) markers. Transplanted LbL-RPE cells remained at the injection site at 5 and 21 wk after surgery (arrowhead). Only a limited number of untreated RPE cells remained in graft area at 5 and 21 wk after surgery (arrowhead). Arrows indicated viable transplanted RPE or LbL-RPE cells which were human mitochondria or RPE65, Hoechst and Dil positive. Scale bar: 50 μm. **Figure S3.** Immunogenicity of RPE cells or LbL-RPE cells In Vivo (A-D) Photomicrographs showed the labeling of RCS rats retinal sections at 5 and 21 wk after transplantation. Anti-Iba1/CD3 antibody (green); many Iba1^+^ cells (arrow) invaded the INL/ONL after RPE transplants, but were poorly labeled after LbL-RPE transplants (RPE and LbL-RPE cells were pre-labeled with Dil (red)). There were numerous CD3^+^ cells (arrow) which infiltrated in the RPE retinas. CD3^+^ cells were very sparse in LbL-RPE transplants. Iba1^+^ cells in the retina were also observed in the control retina section (sham) that injected only with culture medium (without RPE/LbL-RPE cells), but there were no CD3^+^ cells. Scale bars: 50 μm. Supplemental experimental procedures.

## Data Availability

The data used and/or analyzed during the current study are available from the corresponding author on reasonable request.

## Abbreviations

RPE: Retinal pigment epithelial
AMD: Age-related macular degeneration
RCS: Royal College of Surgeons
hESC-RPE: Embryonic stem cell-derived RPE
LbL: Layer-by-layer
IEP: Isoelectric point
TEM: Transmission electron microscope
PEDF: Pigment epithelium-derived factor
POS: Phagocytize photoreceptor outer segments
TER: Transepithelial electrical resistance
PBMCs: Peripheral blood mononuclear cells
MLR: Mixed lymphocyte reaction
ERGs: Electroretinograms
