# Supplementary material for: Improving cell survival and engraftment in vivo via layer-by-layer nanocoating of hESC-derived RPE cells
Source: Stem Cell Res Ther. 2020 Nov 25;11:495. doi: 10.1186/s13287-020-01986-z (PMC7687756; doi:10.1186/s13287-020-01986-z)
Supplement: Supplementary file 1 — Additional file 1: Figure S1. Generation of hESC-RPE cells and effects of layer-by-layer assembly coated on RPE cells (A) A schematic overview of differentiation of hESC into RPE cells. The differentiation from hESC to RPE cells requires three steps: super-confluence, acquired pigment foci, and excision. RPE cells are the cells that diffuse from the excised pigment foci. (B) Calcien AM/PI (propidium iodide) assay showed cell viability of LbL-RPE cells when treated with (gelatin)2/alginate. Lower magnification image was observed by fluorescence microscope. Calcien AM (green) showed viable cells, while PI (red) showed dead cells. (Cell viability = (Total cells - number of dead cells)/Total cells × 100%; cell viability was ~ 95.6%). Scale bar: 200 μm. (C) Growth states of LbL-RPE and untreated RPE cells on days 4, 7, 9, and 18. Scale bar: 200 μm. (D) Phase contrast images of LbL-RPE and untreated RPE cells at 21 and 30 days after differentiation. Scale bar: 100 μm. Figure S2. Survival assessment of RPE and LbL-RPE cells in vivo (A-D) Immunofluorescence staining observed by confocal microscopy. Transplanted cells (pre-labeled with Dil (red)) expressed human mitochondria (green) or RPE65 (green) markers. Transplanted LbL-RPE cells remained at the injection site at 5 and 21 wk after surgery (arrowhead). Only a limited number of untreated RPE cells remained in graft area at 5 and 21 wk after surgery (arrowhead). Arrows indicated viable transplanted RPE or LbL-RPE cells which were human mitochondria or RPE65, Hoechst and Dil positive. Scale bar: 50 μm. Figure S3. Immunogenicity of RPE cells or LbL-RPE cells In Vivo (A-D) Photomicrographs showed the labeling of RCS rats retinal sections at 5 and 21 wk after transplantation. Anti-Iba1/CD3 antibody (green); many Iba1+ cells (arrow) invaded the INL/ONL after RPE transplants, but were poorly labeled after LbL-RPE transplants (RPE and LbL-RPE cells were pre-labeled with Dil (red)). There were numerous CD3+ cells (arrow) which inf [file 13287_2020_1986_MOESM1_ESM.doc]

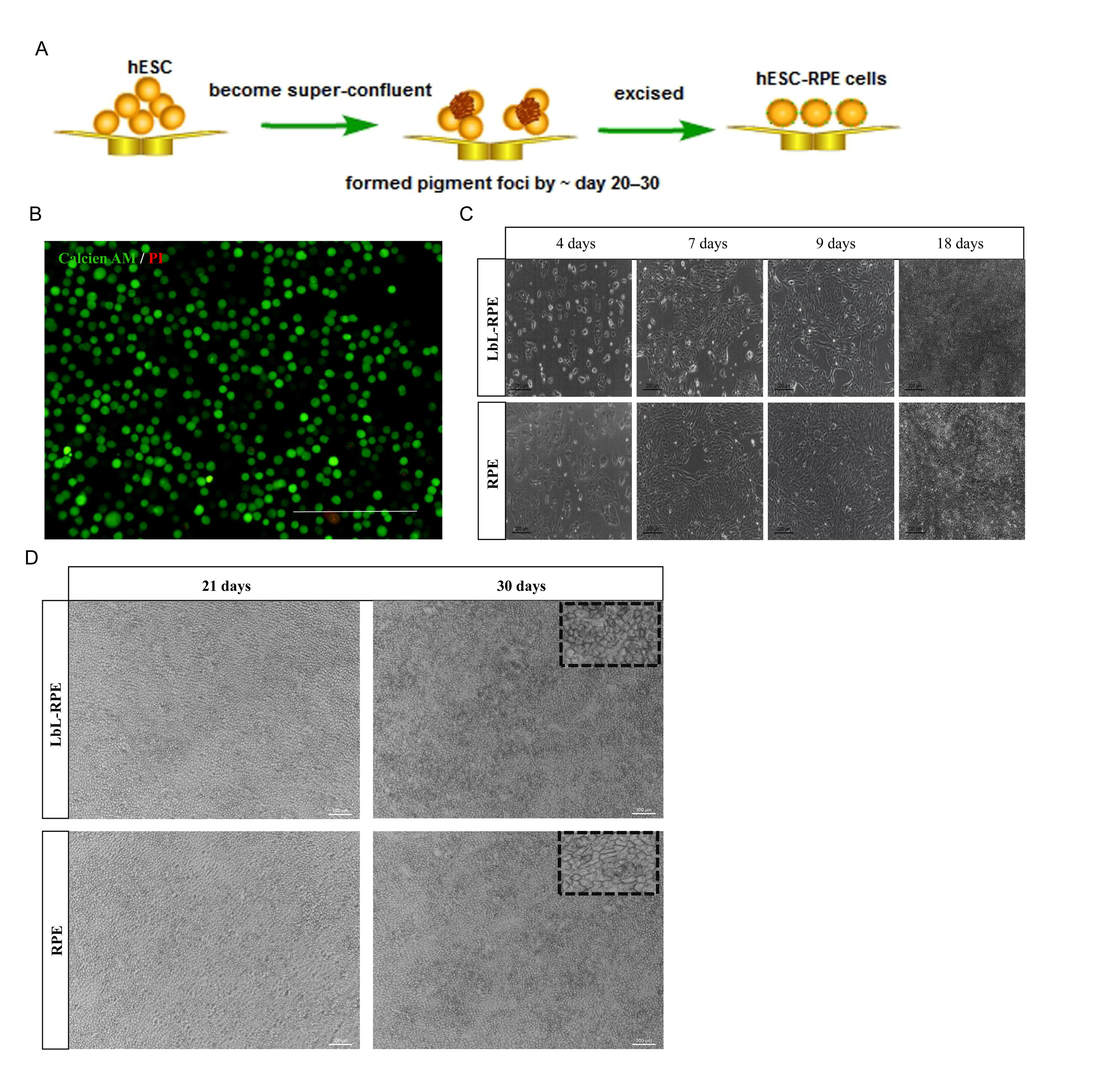


Figure S1. Generation of hESC-RPE cells and effects of layer-by-layer assembly coated on RPE cells
(A) A schematic overview of differentiation of hESC into RPE cells. The differentiation from hESC to RPE cells requires three steps: super-confluence, acquired pigment foci, and excision. RPE cells are the cells that diffuse from the excised pigment foci.
(B) Calcien AM/PI (propidium iodide) assay showed cell viability of LbL-RPE cells when treated with (gelatin)_2_/alginate. Lower magnification image was observed by fluorescence microscope. Calcien AM (green) showed viable cells, while PI (red) showed dead cells. (Cell viability = (Total cells - number of dead cells)/Total cells × 100%; cell viability was ~ 95.6%). Scale bar: 200 μm.
(C) Growth states of LbL-RPE and untreated RPE cells on days 4, 7, 9, and 18. Scale bar: 200 μm.
(D) Phase contrast images of LbL-RPE and untreated RPE cells at 21 and 30 days after differentiation. Scale bar: 100 μm.


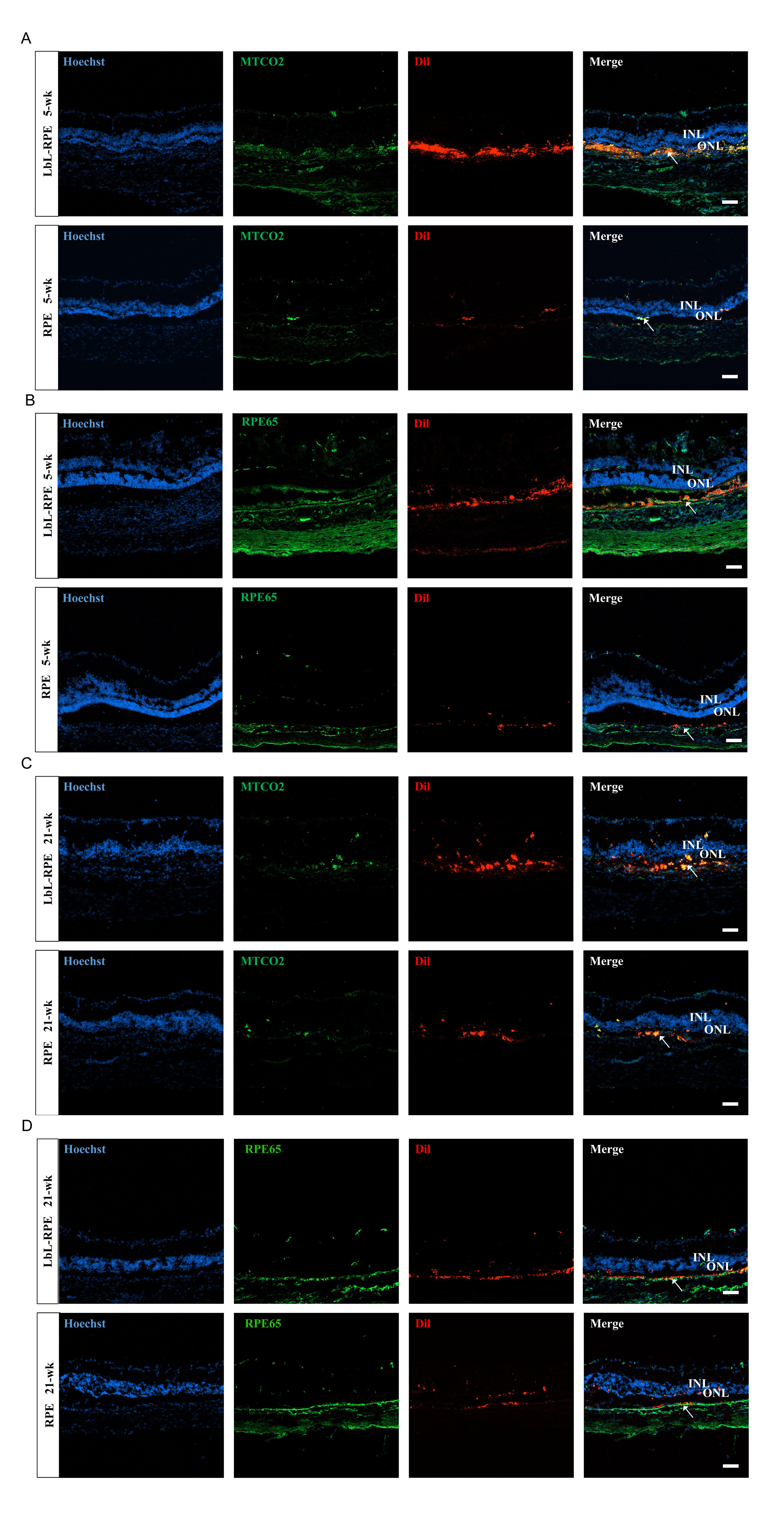


Figure S2. Survival assessment of RPE and LbL-RPE cells *in vivo*(A-D) Immunofluorescence staining observed by confocal microscopy. Transplanted cells (pre-labeled with Dil (red)) expressed human mitochondria (green) or RPE65 (green) markers. Transplanted LbL-RPE cells remained at the injection site at 5 and 21 wk after surgery (arrowhead). Only a limited number of untreated RPE cells remained in graft area at 5 and 21 wk after surgery (arrowhead). Arrows indicated viable transplanted RPE or LbL-RPE cells which were human mitochondria or RPE65, Hoechst and Dil positive. Scale bar: 50 μm.


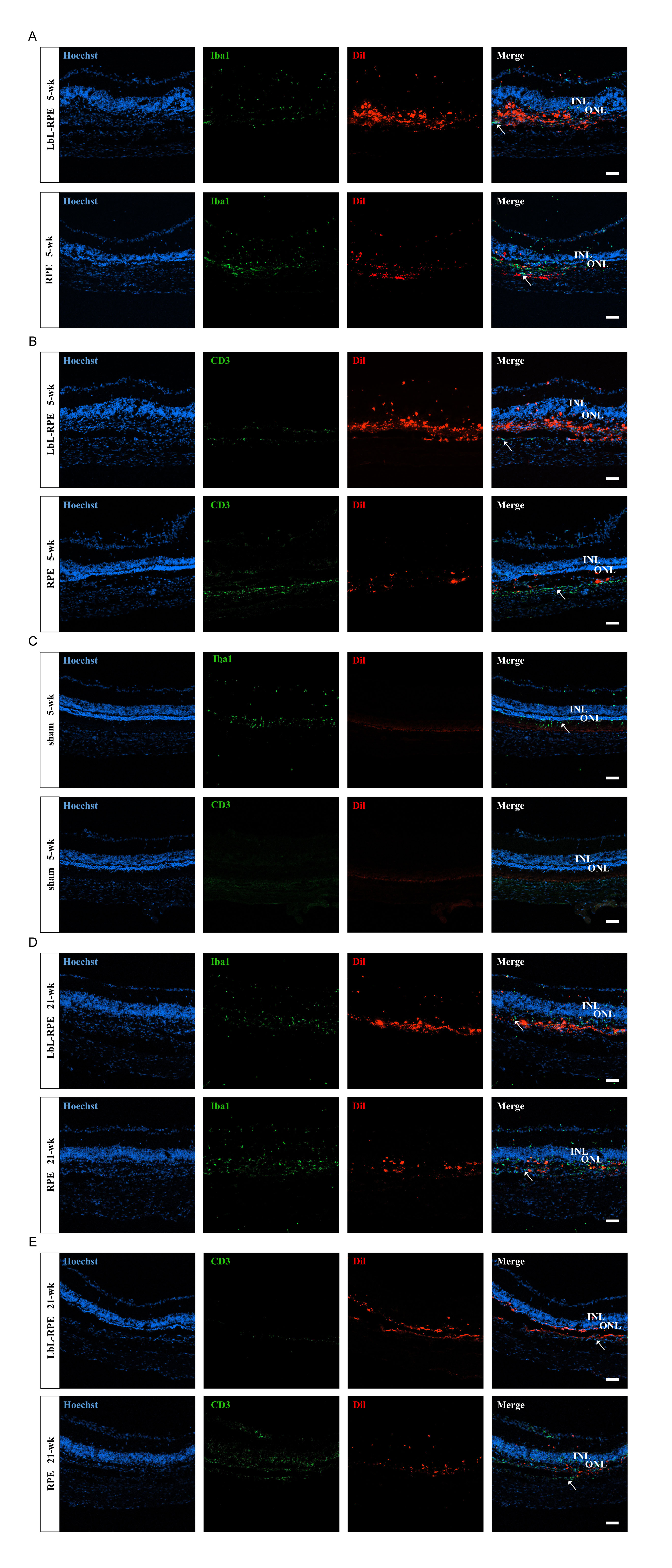


Figure S3. Immunogenicity of RPE cells or LbL-RPE cells *In Vivo*(A-D) Photomicrographs showed the labeling of RCS rats retinal sections at 5 and 21 wk after transplantation. Anti-Iba1/CD3 antibody (green); many Iba1^+^ cells (arrow) invaded the INL/ONL after RPE transplants, but were poorly labeled after LbL-RPE transplants (RPE and LbL-RPE cells were pre-labeled with Dil (red)). There were numerous CD3^+^ cells (arrow) which infiltrated in the RPE retinas. CD3^+^ cells were very sparse in LbL-RPE transplants. Iba1^+^ cells in the retina were also observed in the control retina section (sham) that injected only with culture medium (without RPE/LbL-RPE cells), but there were no CD3^+^ cells. Scale bars: 50 μm.

**SUPPLEMENTAL EXPERIMENTAL PROCEDURES**

*Materials and reagents*: Gelatin (catalog no. G2625), alginate (catalog no. A2158), rhodamine B (catalog no. 283924), ethanediamine (catalog no. 00589), 1-ethyl-3-(3-(dimethylamino) propyl) carbodiimide (EDC; catalog no. E6383), bovine serum albumin (BSA; catalog no. A2153), and propidiumiodide (PI; catalog no. P4170) were obtained from Sigma-Aldrich. Human PEDF ELISA Kit (SerpinF1) (catalog no. ab213815), Anti-Iba1 antibody (catalog no. ab153696), Anti-CD3 antibody (catalog no. ab5690), Anti-Bestrophin/BEST1 antibody (catalog no. ab14929), Anti-RPE65 antibody (catalog no. ab13826), and Aqueous Mounting Medium (catalog no. ab128982) were obtained from Abcam. CTS™ (Cell Therapy Systems) DPBS, without calcium chloride and magnesium chloride (DPBS; catalog no. A1285601), KO-DMEM CTS (catalog no. A1286101), Knock Out SR xenofree CTS (catalog no. 12618013), CTS glutaMAX-1 supplement (catalog no. A12860-01), MEM NEAA (catalog no. 11140-050), CTS™ TrypLE™ Select Enzyme (catalog no. A1285901), Vitronectin, truncated recombinant human (VTN-N; catalog no. A14700), Hoechst 33342 (catalog no. H3570), Anti-ZO-1 antibody (catalog no. 40-2200), Rabbit IgG (H+L) Cross-Adsorbed Secondary Antibody (catalog no. A11008), Mouse IgG (H+L) Cross-Adsorbed Secondary Antibody (catalog no. A11001), Mouse IgG (H+L) Cross-Adsorbed Secondary Antibody (catalog no. A32727), and Cell tracker CM-Dil (catalog no. C700) were obtained from Thermo Fisher Scientific. Fluorescein 5(6)-isothiocyanate (FITC; catalog no. F104848) was purchased from Aladdin, 3-(4,5-Dimethylthiazol-2-yl)-2,5-DiphenyltetraZolium-Bromide (MTT; catalog no. ST316) was purchased from Beyotime and Phallodin. CF488A (catalog no. 00042) was purchased from Biotium and PEDF Antibody FITC Conjugated (catalog no. C00412F) from SAB. Human Recombinant IL-2 (catalog no. BK0240) was purchased from Bioworlde and Recombinant Human IFN-γ Protein (catalog no. 285-IF-100) from R&D systems. All antibodies and corresponding Isotype Controls for flow cytometry were obtained from BD PharMingen.

*Cell Viability Test with Calcien AM/PI Staining*: RPE and (gelatin)_2_/alginate encapsulated RPE cells were prepared, then we followed the experimental instructions of the BB-4126-500T kit to prepare the staining solution. The solution was incubated at 4℃ for 15-30 min, re-suspended in DPBS five times. Finally, images were acquired with a fluorescent microscope (EVOS^TM^, Life technologies) at which time the investigator was blind to the experimental protocol (n = 8).

*Zeta-Potential Assessment*: Gelatin (0.1%) and alginate (0.1%) solutions with 0.01 mol/L HCl, and 0.01 mol/L NaOH were prepared. pH dollars were used to accurately adjust pH between 2 to 10 in 8 steps, and then the zeta-potential determined using a ZEN3690 instrument (Malvern).

*LbL Single-Cell Encapsulation*: A sample of 2×10^6^ RPE cells were centrifuged to remove excess medium then 1 ml of 0.1% gelatin solution added to the tube, and the tube gently shaken for 10 min. Then the tube was centrifuged at 2000 rpm for 5 min, after which, the supernatant was discarded, the cells washed by adding 5 mL Dulbecco’s phosphate-buffered saline (DPBS), the tube again centrifuged, and the supernatant discarded. The cells were incubated for 10 min in 1 ml of 0.1% alginate and the above process repeated in order to coat the cells with three layers of gelatin/alginate/gelatin.

*Methyl thiazolyl tetrazolium Test*: RPE and LbL-RPE cells were cultured at a density of 1×10^4^ per well in 96-well plates coated with VTN-N. Medium was changed every two days. After three days in culture, 10 μL of MTT was added to the wells and the cells incubated for 4 h, after which the solution was removed, followed by the addition of 200 μL of dimethyl sulfoxide (DMSO). Absorbance was examined at 570 nm using a microplate reader (M200PRO, TECAN).

*Preparation of Fluorescent Reagents in Labeled Gelatin and Alginate*: Gelatin-FITC was prepared by dissolving 20 mg of gelatin in 2 mL of 0.1 M sodium bicarbonate buffer to which 10 mg of FITC in 1 mL DMSO was slowly added while magnetically stirring the solution. The solution was incubated overnight at room temperature with continuous stirring then dialyzed for three days and lyophilized. For the alginate-rhodamine B, ethanediamine was used as a bridge. Alginate (10 mg) in 1 mL DPBS was activated by 10 mg EDC for 30 min, then, 3 mg of ethanediamine added, and the mixture stirred overnight at room temperature. The solution was then subjected to dialysis and lyophilization to obtain alginate-ethanediamine powder. The powder was put into 2 mL of rhodamine B solution (2.5 mg/mL in DPBS), and the resultant solution stirred overnight at room temperature. The alginate-rhodamine B was obtained after dialysis and lyophilization.

*Transmission Electron Microscopy (TEM)*: RPE and LbL-RPE cells on glass coverslips were fixed for 1 h in a solution of 2.5% glutaraldehyde diluted with 0.1 M Sorensen’s buffer. Then, the samples were washed for 5 min with 5% sucrose in 0.1 M Sorensen’s buffer (3x) and stored at 4℃ overnight. Post fixation included 1% OsO4 for 2 h then the cells were dehydrated in graded alcohols and methanol, and finally embedded in epoxy resin (60℃ for 24 h). A microtome (UC7 RT, Leica) was used to obtain thin sections, which were then mounted on copper grids, stained with 2% uranyl acetate and 1% lead citrate and analyzed with a TEM (JEM-1400, JEOL).

*Scanning Electron Microscopy (SEM)*: After 7 days of culture, RPE and LbL-RPE cells were fixed for 2 h in 0.1 M Sorensen’s buffer containing 2.5% glutaraldehyde. Then, the samples were rinsed three times (5 min each) with 0.1 M Sorensen’s buffer containing 5% sucrose, post-fixed in 1% OsO4 for 2 h at 4℃, followed by three cycles of washing, then dehydrated with graded alcohols (20%, 40%, 50%, 75%, 90%, 100%) and dried. Samples were sprayed with gold prior to observation with a SEM (S-3400N II, Hitachi).

*Preparation of photoreceptor rod outer segment (POS)*: Retinal tissue from two rat eyeballs was isolated and placed in a 3 mL solution of 20 mmol/L Tris-HCl in a 5 mL centrifuge tube, centrifuged at 6000 rpm for 5 min at 4℃ and the supernatant discarded. The retinal tissue was washed twice by adding 500 μL of Tris-HCl to a 1.5 mL tube, centrifuged at 15000 rpm for 30 min at 4℃, and POS deposition collected.

*Phagocytosis assay*: Phagocytosis was assessed by a flow cytometry-based method using rod outer segment-FITC. POS-FITC was prepared by dissolving POS in 10 ml 0.1 mol/L carbonate buffer solution, to which 0.006 g FITC was slowly added. The reaction mixture was gently shaken for 1 h at room temperature in the dark, then the solution centrifuged at 15,000 rpm for 25 min, after which, the supernatant was discarded. Cells were washed by adding 5 mL Dulbecco’s DPBS, then the tube again centrifuged and the supernatant discarded. POS-FITC deposition was then re-suspended in 100 μL of culture medium. RPE and LbL-RPE cells were incubated with POS-FITC in an incubator for 3 hours at 37℃. Cell cultures were examined under the microscope, harvested by CTS™ TrypLE™ Select Enzyme, and counted by flow cytometry (10,0000 events).

*Transepithelial electrical resistance (TER) measurements*: Untreated RPE and LbL-RPE cells were seeded at 3×10^5^ cells/cm^2^ on 1-µm pore transwell filters (Millipore). TER was measured from day 3 to day 30 using an EVOM epithelial voltohmmeter (World Precision Instruments, Hamden). Prism 6 was used to analyze the TER of the six transwell filters with untreated RPE or LbL-RPE cells, and the control was the empty transwell filters only coated with Vitronectin, truncated recombinant human (VTN-N).

*Preparation of Human PBMCs and T Cells*: After informed consent was obtained, human peripheral blood mononuclear cell (PBMC) cultures were established from human peripheral blood using Ficoll-Paque PLUS (HY2015, TBD). CD4^+^ T cells were prepared separately by using separation beads (Human CD4 T Lymphocyte Enrichment Set-DM, 557939, BD PharMingen). These cells were more than 88% CD4-positive. T-cells were co-cultured with RPMI-1640 medium containing 10% human AB serum, human recombinant IL-2 (10 ng/ml), 10 mM HEPES, 0.1 mM nonessential amino acids, 1 mM sodium pyruvate, penicillin-streptomycin, and 2-mercaptoethanol.

*ELISA Assay*: A standard or samples were added to a microplate coated with anti-PEDF or anti-IFN-γ (60 min), washed in buffer three times, then 100 μL of prepared ABC working solution added to each well and incubated for 30 min, washed again, and a prepared TMB color developing agent pipetted into the plates and incubated for a further 25-30 minutes in the dark. Immediately after adding the stop solution, a microplate reader (M200PRO, TECAN) was used to determine the optical density at 450 nm.

*Mixed Lymphocyte Reactions with RPE and LbL-RPE cells*: PBMCs were isolated from 9 healthy donors and co-cultured with RPE and LbL-RPE cells (effector/target ratio = 10:1) for 96 ~ 120 hr. After 96 ~ 120 hr, PBMCs culture medium was collected, and immune responses were assessed by IFN-γ production using ELISA (Human IFN-γ ELISA Kit II, BD, 550612) (*p < 0.05). Data represent the mean ± SEM of three independent experiments.

*Animal Experiments*: The care and maintenance of the rats conformed to the Vision and Ophthalmology Statement for the Use of Animals in Ophthalmic and Vision Research, and the Use of Laboratory Animals, as well as the Guidelines of IACUC of the Southwest Hospital. The RCS rats used in this study were maintained in the animal facility of the Southwest Hospital, the Third Military Medical University under standard laboratory conditions (18–23℃, 40%–65% humidity, 12 hr light/12 hr dark cycle) with adequate food and water.

*Full-field ERG recordings*: Full-field electroretinograms (ERGs) were recorded after overnight dark adaptation. ERGs were recorded 2 and 5 wk after transplantation from both transplant groups (n = 8 rats per condition), and a Sham group (n = 8). The animals were anesthetized with an intraperitoneal injection of 1% pentobarbital sodium. The corneas were anesthetized with a drop of 0.5% proparacaine hydrochloride, and the pupils dilated with 1% tropicamide. The animals at 2 wk after transplantation were allowed to recover and ERGs again recorded at 5 wk. Recording electrodes were placed on the cornea. Responses were differentially amplified following stimulation with light pulses of 3.2×10^-5^ - 10 cds/m^2^ and measurements from six flashes averaged. B-wave amplitude was determined from the trough of the a-wave to the peak of the b-wave. ERGs were recorded at 21 wk after transplantation from both transplant groups which showed unrecorded amplitudes.

*Immunofluorescent Staining*: To identify F-actin of cells and pigment epithelium-derived factor (PEDF) secretion from RPE cells or LbL-RPE cells, and study the expression of typical RPE cell markers in RPE or LbL-RPE cultured cells, cells were grown on cover slips, fixed in 4% paraformaldehyde for 15 min at 4℃, rinsed three times (5 min each) with DPBS, and incubated in 0.5% TritonX-100 in DPBS for 10-15 min to permeabilize the cells. Cells were rinsed three times (5 min each) with DPBS, incubated with 1% BSA in DPBS for 30 min to block unspecific binding, and then incubated with primary antibodies in DPBS containing 1% BSA for 30 min-1h at 37℃. The cells were rinsed with DPBS three times (5 min each) in the dark and stained with Hoechst 33342 (1:2500 in DPBS) for 5 min in the dark, and again rinsed with DPBS. Finally, the cells were coversliped with a drop of mounting medium. The primary antibodies used were as follows: PEDF Antibody FITC Conjugated (1:50-1:200), Phallodin CF488A (1:40), ZO-1 (1:100), RPE65 (1:50-100), and BEST1 (1:400). To evaluate the distribution of microglial cells, T lymphocytes, and RPE cell survival in the retina of RCS rats (5 weeks and 21 weeks post-transplant), frozen retinal sections (12 µm thick) were stained with rabbit anti-Iba1 (1:500), rabbit anti-CD3 (1:100), rabbit anti- MTCO2 (1:200), and Mouse anti-RPE65 (1:50-100). Sections were blocked in PBS containing 10% of normal goat serum and 0.5% of Triton for 10-15 min at room temperature and then incubated with the primary antibodies overnight at 4℃. Rabbit IgG (H+L) Cross-Adsorbed Secondary Antibody (A11008, Invitrogen, 1:500), Mouse IgG (H+L) Cross-Adsorbed Secondary Antibody (A11001, Invitrogen, 1:2000), and Rabbit IgG (H+L) Cross-Adsorbed Secondary Antibody (A32731, Invitrogen, 1:200-2000) were used as secondary antibodies. Images were acquired with a confocal microscope (LSM 800, ZEISS) in a blind study manner.

*Cell Counting*: For the number of gelatin/alginate/gelatin- FITC encapsulated RPE cells, standardized images were taken under the microscope of whole eye sections stained with FITC and Hoechst. The number of gelatin/alginate/gelatin- FITC encapsulated RPE cells was determined by counting cells co-staining with FITC and Hoechst in the grafted area. Three areas of one retinal section and three retinal sections of each eye were counted and 3 eyes per group were included.

For quantification of MTCO2^+^, RPE65^+^, Iba1^+^, and CD3^+^ cells in the retina were counted from standardized images that were taken under the microscope as above. In all cases, co-staining with Hoechst was used to mark all cells in the retina and for determining the relative location of counted cells in the retina. Cell counts include 3-6 independent sections per eye. Three to eight eyes per group were included.
